# A novel prognostic prediction model of cuprotosis-related genes signature in hepatocellular carcinoma

**DOI:** 10.3389/fcell.2023.1180625

**Published:** 2023-08-07

**Authors:** Ruo-Nan Shao, Kun-Hao Bai, Qian-Qian Huang, Si-Liang Chen, Xin Huang, Yu-Jun Dai

**Affiliations:** ^1^ State Key Laboratory of Oncology in South China, Collaborative Innovation Center for Cancer Medicine, Guangzhou, China; ^2^ Department of Hematologic Oncology, Sun Yat-sen University Cancer Center, Guangzhou, China; ^3^ Department of Endoscopy, Sun Yat-sen University Cancer Center, Guangzhou, China; ^4^ Department of Hematology, Peking University Shenzhen Hospital, Shenzhen, China; ^5^ Department of Pancreatobiliary Surgery, Sun Yat-sen University Cancer Center, Guangzhou, China

**Keywords:** cuprotosis (CRGs), prognostic model, hepatocellular carcinoma (HCC), treg cells, immunotherapy

## Abstract

**Background:** Cuprotosis is a recently discovered copper-dependent cell death mechanism that relies on mitochondrial respiration. However, the role of cuprotosis-related genes (**CRGs**) in hepatocellular carcinoma (**HCC**) and their prognostic significances remain unknown.

**Methods:** Based on the recently published **CRGs**, the LASSO Cox regression analysis was applied to construct a **CRGs** risk model using the gene expression data from the International Cancer Genome Consortium as a training set, followed by validation with datasets from The Cancer Genome Atlas and the Gene Expression Omnibus (GSE14520). Functional enrichment analysis of the **CRGs** was performed by single-sample gene set enrichment analysis.

**Results:** Five of the 13 previously published **CRGs** were identified to be associated with prognosis in HCC. Kaplan-Meier analysis suggested that patients with high-risk scores have a shorter overall survival time than patients with low-risk scores. ROC curves indicated that the average **AUC** was more than 0.7, even at 4 years, and at least 0.5 at 5 years. Moreover, addition of this **CRG** risk score can significantly improve the efficiency of predicting overall survival compared to using traditional factors alone. Functional analysis demonstrated increased presence of Treg cells in patients with high-risk scores, suggesting a suppressed immune state in these patients. Finally, we point to the possibility that novel immunotherapies such as inhibitors of *PDCD1, TIGIT, IDO1, CD274, CTLA4,* and *LAG3* may have potential benefits in high-risk patients.

**Conclusion:** We constructed a better prognostic model for liver cancer by using **CRGs**. The **CRG** risk score established in this study can serve as a potentially valuable tool for predicting clinical outcome of patients with **HCC**.

## Background

Multicellular organisms have a variety of predetermined and precisely programmed cell death pathways, such as apoptosis, necroptosis (programmed necrosis), pyroptosis (inflammation mediated), and ferroptosis (iron regulated cell death) (Yan et al., 2022). Recent research reported a novel mechanism known as cuprotosis where cell death is regulated by copper. This mechanisms can be triggered by copper ions even when other common cell death pathways are blocked (Tang et al., 2022). Copper ions directly bind to fatty acylated components of the tricarboxylic acid (**TCA**) cycle within the mitochondria, leading to aggregation of fatty acylated proteins and downregulation of iron-sulfur cluster proteins, which induces proteotoxic stress and cell death (Tsvetkov et al., 2022). This novel pathway may have significant implications for understanding cancer biology and treatment.

Copper concentrations are elevated in the tumor tissues and serum samples of animals and patients with cancers (Jiang et al., 2022). The level of copper is associated with liver cirrhosis, acute hepatitis, and liver cancer. Serum copper may be useful as a marker for liver cancer detection (Jaafarzadeh et al., 2021). In patients with hepatocellular carcinoma (**HCC**), excessive copper concentrations can enhance tumor development, chemoresistance, and poor prognosis (Fang et al., 2019). All the above studies indicate that copper may be related to the occurrence of liver tumors, providing a new perspective for the treatment of this malignancy (Ge et al., 2022).

Here, we comprehensively explored the clinical relevance of the expression of cuproptosis-related genes (**CRGs**), their molecular alterations, and the tumor immune microenvironment in **HCC**. Moreover, our study also constructed a new prognostic model for **HCC** with **CRGs** and laid a foundation for potential therapeutic development utilizing cuproptosis regulators for **HCC** targeting and immunotherapy.

## Methods

### Data acquisition

Gene expression information and related clinicopathologic data of 817 HCC patients were retrieved from The Cancer Genome Atlas (https://portal.gdc.cancer.gov/repository) (**TCGA**, 231 samples), International Cancer Genome Consortium (https://dcc.icgc.org) (**ICGC**, 231 samples) and Gene Expression Omnibus (http://www.ncbi.nlm.nih.gov/geo/) (**GEO**, GSE14520, 365 samples). Log2 transformation was performed to normalize the expression profiles of the gene sets. A total of 370 samples with copy number variation (**CNV**) and single nucleotide variant (**SNV**) relevant to HCC were downloaded from the **TCGA-LIHC** site (University of California Santa Cruz Xena database). Moreover, 13 **CRGs** were collected from a previous literature (Tsvetkov et al., 2022) and are shown in Supplementary Table S1.

### Cuproptosis-related prognostic signature model

**FIGURE 1:**
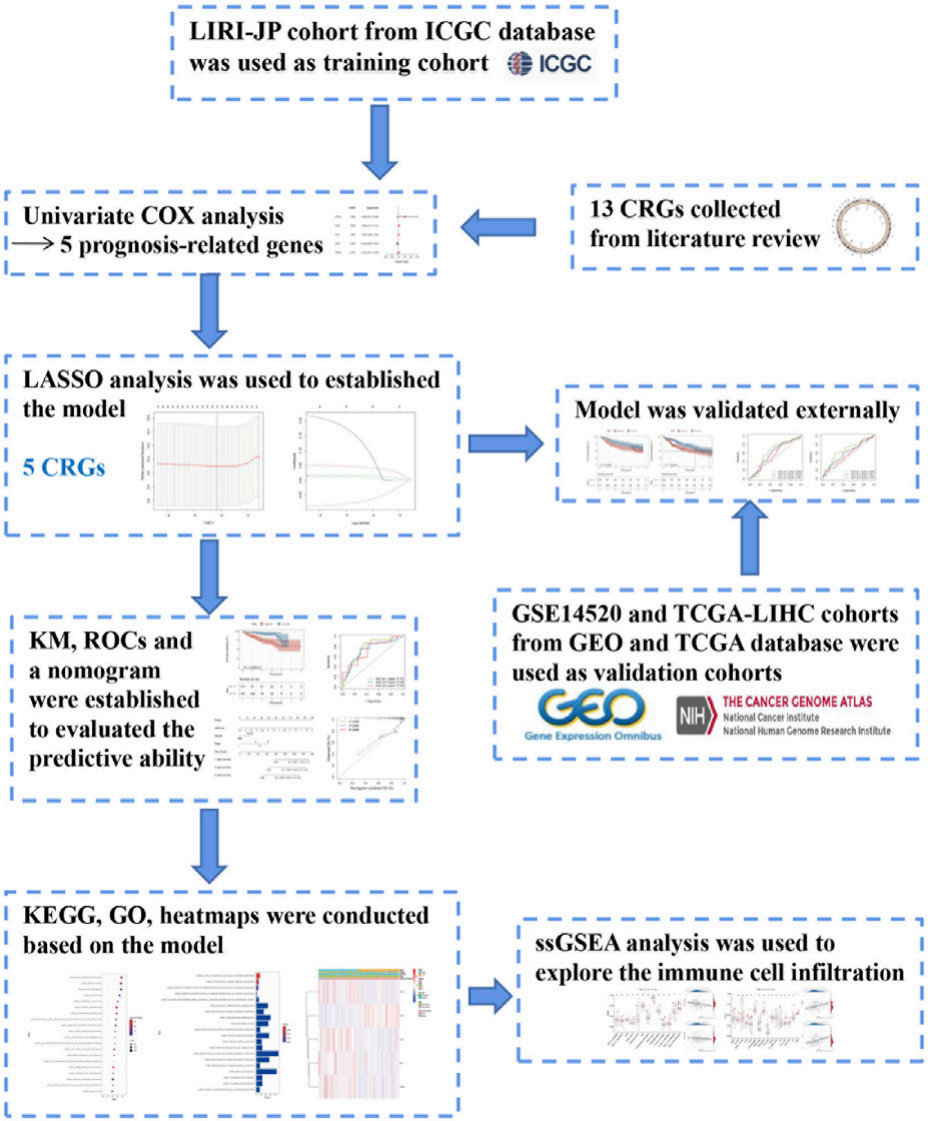
Flow chart of data collection and analysis, LIRI-JP cohort OCHC database was used as training cohort.

The LIRI-JP cohort from the **ICGC** database was employed as the training cohort. Overall survival (**OS**)—related **CRGs** were screened via the univariate Cox analysis (*p* < 0.1). The prognostic **CRG** signature was constructed using the LASSO regression analysis based on 10-fold cross-validation penalized maximum likelihood estimators. The minimum criteria were used to choose the optimal penalty parameter (λ) values. The GSE14520 and **TCGA-LIHC** datasets were selected as the external validation cohorts. We calculated the **CRG** risk score (**RS**) for each **HCC** patient using the following formula: RS = (β * *ATP7A* expression level) + (β * *DLAT* expression level) + (β * *DLD* expression level) + (β * *FDX1* expression level) + (β * *PDHB* expression level), where β is the coefficient for each gene. Patients were further assigned into the high- and low-risk sets in accordance with the median RS. Kaplan-Meier and time-dependent receptor operating characteristic (**ROC**) curves were employed to assess the predictability of the **CRG** signature. The design of the study is shown in Figure 1.

### Cell lines

The liver cancer cell lines including HEG2, MHCC97-H, HUH-7, SNU449, PLC-PRF-5, LM3, and LM9, and normal liver cell lines such as HL7702, WRL68, QSG-7701, and MIHA cells were obtained from Sun Yat-sen University Cancer Center. The expression data of these **CRGs** were obtained from Cancer Cell Line Encyclopedia (**CCLE**).

### Quantitative reverse transcription polymerase chain reaction (qRT-PCR)

Total RNA was extracted from cells using TRIzol reagent (Takara Bio, Carlsbad, United States) and reverse transcribed using a cDNA reverse transcription kit (Takara Bio, Carlsbad, United States) in accordance with the manufacturer’s instructions, and the obtained cDNA was amplified using TB Green^®^ Premix Ex Taq (Takara Bio, Carlsbad, United States). qRT-PCR was performed to detect expression levels of the genes of interest. Each experiment was repeated three times. The 2^−ΔΔCT^ methodology was adopted to calculate the relative expression of genes. The primers used are listed in Supplementary Table S2.

### Functional enrichment analysis

The GSEA_4.2.3 software was applied to examine the physiological pathways that genes in the low- and high-risk datasets are involved in according to the KEGG and GO analyses, “c2. cp.kegg.v7.5.1. symbols” and “c5. go.bp.v7.5.1. symbols”, respectively. Normalized *p*-value <0.05 was considered statistically significant. In addition, we calculated the activity of 13 immune-linked networks and 16 immune cell types through the single-sample gene set enrichment analysis (**ssGSEA**) (Rooney et al., 2015). Protein interactions between model-related proteins were constructed with the STRING algorithm (https://cn.string-db.org). Genetic variation information in the cancer cell lines was from the cBioPortal Genomics database. DNA methylation analysis was performed by methsurv (https://biit.cs.ut.ee/methsurv/) (Modhukur et al., 2018; Anuraga et al., 2021; Xing et al., 2021).

### Statistical analysis

The Student’s t-test or Wilcoxon test was employed to analyze continuous data. **OS** comparisons between two sets were completed by the log-rank test. The time-**ROC** package was applied to complete the ROC curves and estimate the values of the area under the curve (**AUC**). The independent prognosis index was estimated by the uni- and multivariate COX analyses. All statistical analyses were performed using the R software (Version 4.0.4) or SPSS (Version 25.0). A two-sided *p*-value <0.05 indicated statistical significance.

## Results

### Genetic landscape of cuprotosis related genes

**FIGURE 2:**
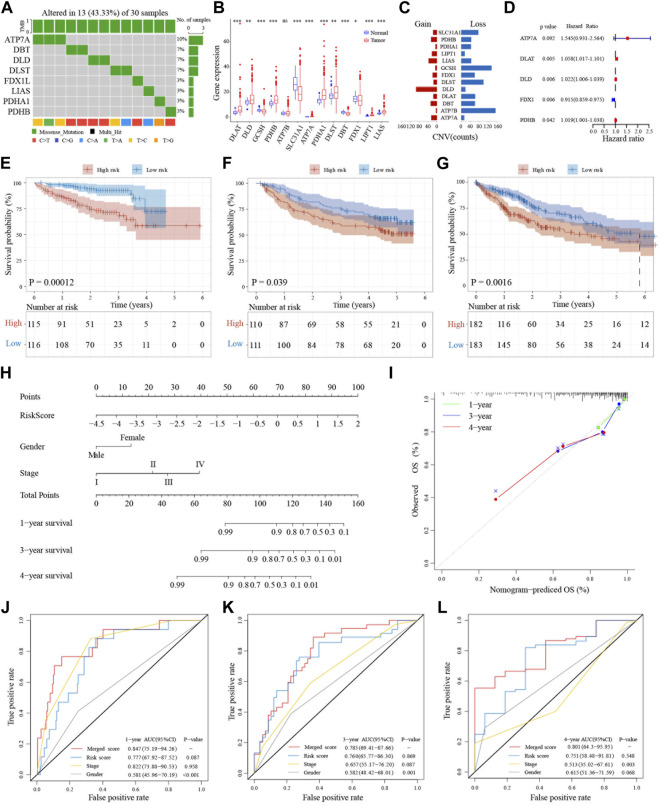
Genetic landscape and prognostic significance of CRGs in HCC. **(A)** Mutation status of 13 CRGs in the TCGA database. **(B)** Tumor-normal expression difference of CRGs in TCGA database. **(C)** CNV situation of CRGs in TCGA database. **(D)** Forest plot of five prognosis-related CRGs in ICGC database. **(E–G)** Kaplan-Meier curves for the OS of patients in the high-risk group and low-risk group in ICGC, GSE14520 and TCGA cohort. **(H)** Nomogram model built on the ICGC dataset. **(I)** Calibration curves for nomogram models. **(J–L)** AUC of time-dependent ROC curves verified the prognostic performance of merged risk score in 1-year, 3-years or 4 years of ICGC, GSE14520 and TCGA cohort.

A recent study reported 13 genes related to the cuproptosis pathway, including *ATP7A*, *ATP7B*, *DBT*, *DLA*, *DLD*, *DLAT*, *DLST*, *FDX1*, *GCSH*, *LIAS*, *LIPT1*, *PDHA1*, and *PDHB* (Tsvetkov et al., 2022). To determine whether these cuproptosis-related genes (**CRGs**) are involved in **HCC**, we extracted their expression levels from 817 **HCC** patient samples from three databases (**TCGA**, **ICGC** and **GEO**) for further analysis (Supplementary Figure S1A; Supplementary Table S1). Many of these **CRGs** are mutated in **HCC** samples and the top 10 mutated genes with the highest frequencies are showed in the Supplementary Figure S1A. Among them, the gene with the highest mutation frequency is *ATP7A*, accounting for about 10%, followed by *DLST*, *DLD*, and *DBT* accounting for about 7%. The major mutation type is missense mutation (43.33%, 13/33), with C>T being the most common (Figure 2A). The expression levels of most CRGs, except for *FDX1*, showed a positive correlation to **HCC** samples (Supplementary Figure S1B). In addition, the **CRGs**, *DLAT*, *DLD*, *PDHB*, *ATP7A*, *PDHA1*, *DLST*, *LIPT1*, and *LIAS*, are also expressed at significantly higher levels in liver cancer cells than in normal tissues (Figure 2B). On the other hand, the heatmaps suggested that expression of *ATP7A*, *DBT*, and *LIPT1* are lower than other genes, and lower in tumors compared to controls (Supplementary Figure S1C). Twelve of the **CRGs** are significantly differentially expressed in the **TCGA** database and analysis also indicated that *FDX1* has the lowest expression (Supplementary Figure S1D). In addition, except for *ATP7A* and *PDHA1*, which are located on the X chromosome, all other genes are located in the autosomes (Supplementary Figure S1E). Copy number variation (**CNV**) analysis showed that most of the 13 genes have copy number losses, with *GCSH* and *ATP7B* being the most obvious, while *DLD* showed a copy number gain (Figure 2C). We further validated the expressions of the **CRGs** in liver cancer cell lines and related normal cells and found that the expression of *DLAT* and *DLA* are much higher and *FDX1* lower in cancer cell lines compared to normal cells (Supplementary Figure S2A). We also validated the same results of CRGs expressions in **HCC** cancer cell lines through Cancer Cell Line Encyclopedia (**CCLE**) project (Supplementary Figure S2B).

### Establishment and validation of a prognostic model for HCC

Next, we used the **ICGC** databset to explore the prognostic value of these 13 **CRGs** in liver cancer. The forest plot results indicated that the expressions of five genes (*ATP7A*, *DLAT*, *DLD*, *FDX1*, and *PDHB*) are associated with prognosis. Except for *FDX1*, expressions of the other four genes are closely related to poor prognosis (Figure 2D). The gene correlation results also pointed out that in addition to *FDX1*, the other **CRGs** are associated with at least three or more other genes (Supplementary Figure S3A). Protein interaction analysis showed that FDX1 is weakly associated with the other proteins, while DLD, PDHB, and DLAT have stronger interactions among these five proteins (Supplementary Figure S3B). Moreover, the mutational landscape of these five **CRGs** in different cancer cell lines indicated that they also have different frequencies of mutations in tumor cells (Supplementary Figure S3C). Further, LASSO-Cox regression analysis of these five prognosis-related **CRGs** in the **ICGC LIRI-JP** training dataset showed that they can be used as a cuprotosis signature (Supplementary Figures S3D, E).

To further examine the prognostic significance of this five-gene cuprotosis signature in **HCC**, we validated this signature in the GSE14520 and **TCGA** datasets. A **CRG** risk score was established using the expression levels of the five **CRGs** and the **HCC** patients were divided into two groups based on the median **CRG** risk score. Patients in different risk categories are scattered in two directions (Supplementary Figures S4A–C). The scatter charts demonstrated that patients with high-risk scores have shorter survival time than patients with low-risk scores (Supplementary Figures S4D–F). This can also be seen in the Kaplan-Meier analysis showing that high-risk patients have shorter overall survival than low-risk patients in both the training and validation datasets (Figures 2E–G). To further validate the survival prediction of this prognostic CRG signature, we utilized the time-dependent **ROC** curves to analyze the **AUC** between the specificity and sensitivity of these risk factors in liver cancer patients. In the training set, the **AUC** was more than 0.7, even at 4 years, and it was also at least 0.5 at 5 years in the validation datasets (Supplementary Figures S4G–I).

## Implications of the CRG risk score for clinical features and prognosis

To further validate the importance of the **CRG** risk score in clinical features and prognosis, univariate and multivariate analyses were applied to examine whether the **CRG** risk score can be an independent prognostic marker for **OS** in **HCC** patients. Univariate Cox analysis showed that a high-risk score is a poor prognostic indicator of OS in liver cancers (Supplementary Figures S5A–C). Moreover, when combining with other well-known prognostic factors, multivariate Cox analysis suggested that the **CRG** risk score can also be a significant predictor of **OS** in liver cancer (Supplementary Figures S5D–F). Further, heatmap of clinical features including grade, TNM staging, AFP levels, BCLC staging, ALT levels, HBV status, and so on indicated that some of these biomarkers distributed differently in the high- and low-risk groups (Supplementary Figures S5G–I).

To further expand the clinical applicability of the five-**CRG** signature, a nomogram of clinical variables and the **CRG** risk score was created as shown in Figure 2H. A total score was obtained for each patient by combining the scores for each prognostic criterion. The results suggested that patients with higher total scores have poorer clinical outcomes. Furthermore, the nomogram calibration plots are highly consistent with the operating modes of the ideal model and predicted the 1-, 3- and 4-year survival time (Figure 2I). The **AUC** for 1-year overall survival of the merged score group is 0.847 [95% CI: 0.75–0.94], the **CRG** risk score group is 0.777 [95% CI: 0.68–0.88], the stage is 0.822 [95% CI: 0.74–0.91], and the gender is 0.581 [95% CI: 0.46–0.70]. In addition, the **AUC** for 3-year survival of the merged score group is 0.785 [95% CI: 0.69–0.88], the **CRG** risk score group is 0.760 [95% CI: 0.66–0.86], the stage is 0.657 [95% CI: 0.55–0.76], and the gender is 0.582 [95% CI: 0.48–0.68]. Further, the **AUC** for 4-year OS of merged score group is 0.801 [95% CI: 0.64–0.96], the **CRG** risk score group is 0.751 [95% CI: 0.58–0.92], the stage is 0.513 [95% CI: 0.35–0.68], and the gender is 0.615 [95% CI: 0.51–0.72]. All these results suggested that the addition of this five-**CRG** risk score can significantly improve the **OS** prediction efficiency compared to traditional factors alone (Figures 2J–L).

### Functional analyses of the CRG risk model

**FIGURE 3:**
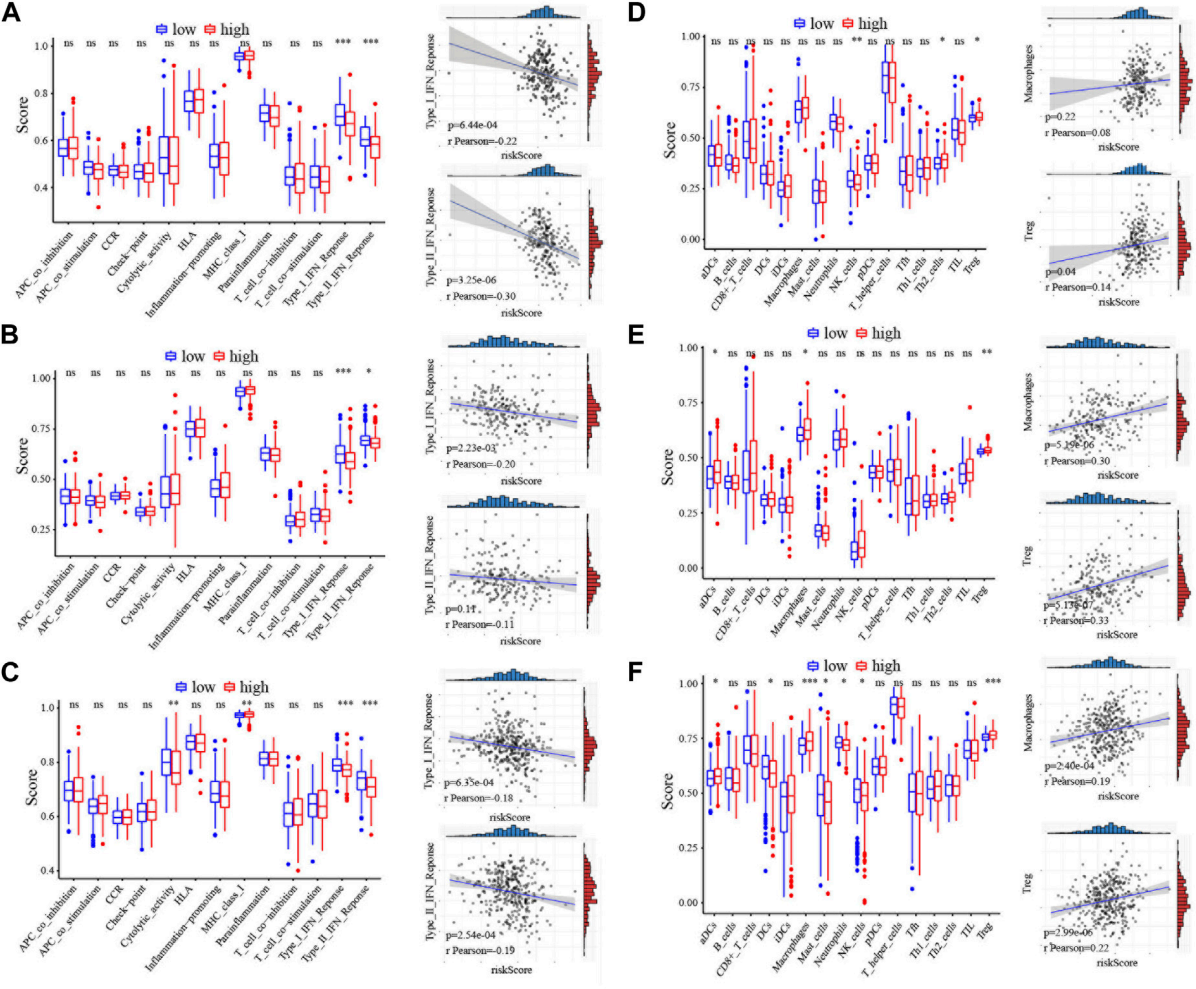
Immunoassay correlation analysis of CRGs in HCC. **(A–C)** Immune-related functions between different risk groups in ICGC, GSE14520 and TCGA cohort. The correlation of the type I IFN response or type II IFN response with risk score was displayed on the right panel. The relation value was calculated by pearson analysis. **(D–F)** The scores of immune cells between different risk groups in ICGC, GSE14520 and TCGA cohort. The correlation of the macrophages or Treg cells with risk score was displayed on the right panel. The relation value was calculated by pearson analysis. *, *p* < 0.05; **, *p* < 0.01; ***, *p* < 0.001.

Since the five-**CRG** signature described above can distinguish between high- and low-risk patients, we look wider to asked which genes are differentially expressed between these patient subgroups. We applied “limma” to identify the differentially expressed genes with the criterion (|log_2_FC| ≥ 1 and FDR <0.05) in the **ICGC**, GSE14520 and **TCGA** datasets. Functional pathway analysis of these differentially expressed genes using Go terms showed that immune response pathways of different types of immune cells are more enriched in the high-risk score group. Moreover, single sample gene set enrichment analysis (**ssGSEA**) functional results further indicated that several different immune-related pathways are closely associated with the **CRG** risk score (Supplementary Figures S6A–C). What caught our attention was that the results of the refined immunophenotyping analysis suggested that the type I and II interferon (**IFN**) response pathway is the only pathway significantly more enriched in the low-risk score group in all three datasets (Figures 3A–C). To further determine the correlation between immune cell infiltration and the **CRG** risk score, we quantified and analyzed the enriched fractions of different immune cell subsets using ssGSEA. We found that NK cells, Th2, and Treg cells have significant differences between the high- and low-risk groups in the **ICGC** dataset. In the GSE14520 dataset, activated dendritic cells (**aDCs**), macrophages, and Treg cells are more enriched in the high-risk group. While in the **TCGA** dataset, aDCs, DCs, macrophages, neutrophils, masts, NK cells, and Treg cells have significant enrichment differences (Figures 3D–F). Interestingly, Treg cells are the only immune cell subtype, that is, more enriched in the high-risk score group, with significant differences in all three datasets.

### CRG-related immune microenvironment and therapeutic targets

**FIGURE 4:**
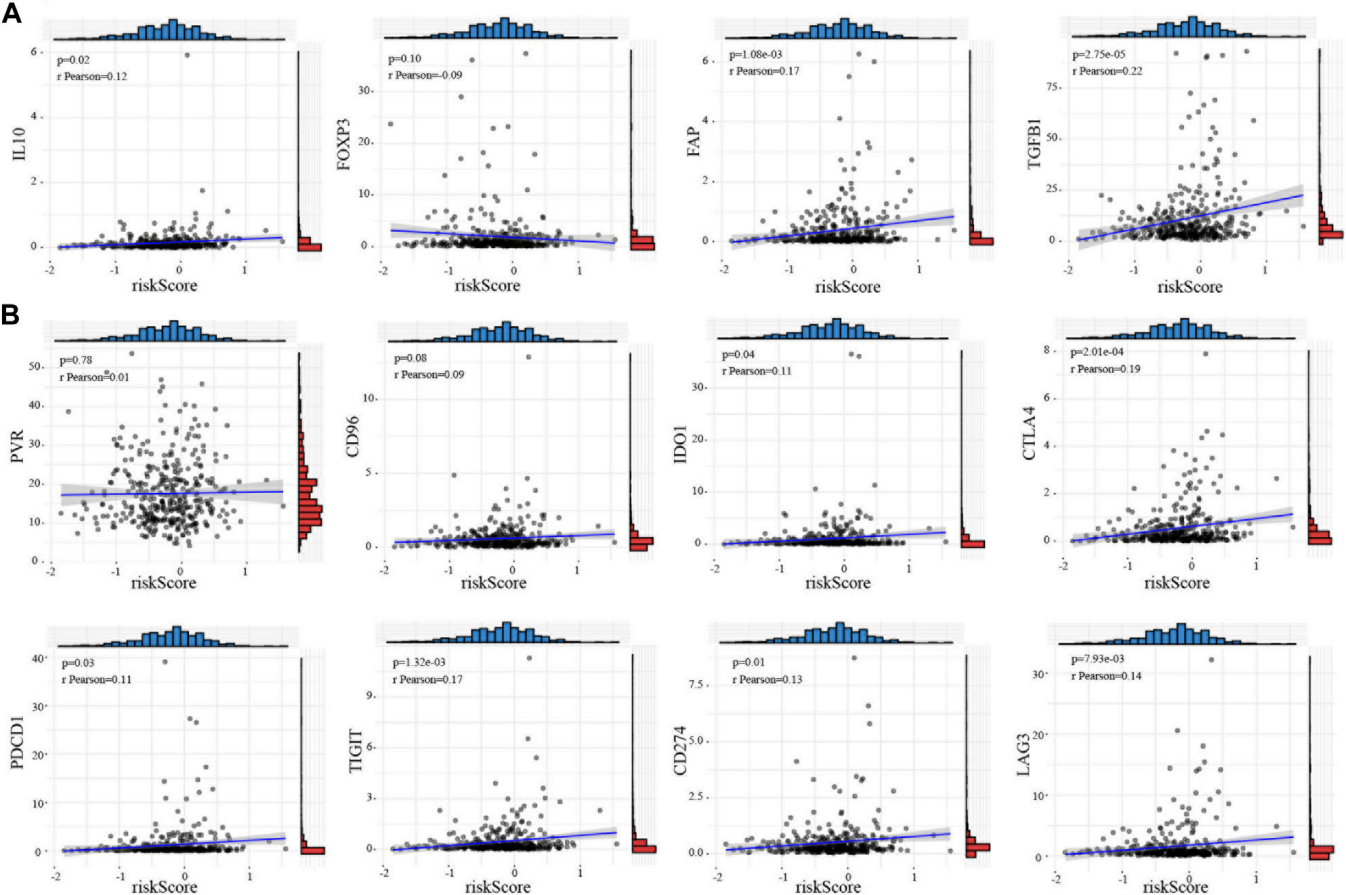
Immune checkpoint target correlation analysis of CRGs in HCC **(A)** Relationship between the risk score and immunosuppressive marker molecules including *IL-10, FOXP3, FAP, TGFB1*, and *IL-6*. **(B)** The correlation between the risk score and the targets of immunotherapy such as *PVR, PDCD1, CD96, TIGHT, IDO1, CD274, CTLA4*, and *LAG3*. The relation value was calculated by pearson analysis.

Cancer immunotherapy has made great breakthroughs and significantly improved the survival rate of cancer patients (Riley et al., 2019). Our results showed that the high-risk score is closely associated with Treg cells, indicating that cuprotosis may affect the prognosis of **HCC** patients by regulating the tumor immune microenvironment. We explored the relationship between the **CRG** risk score and immunosuppressive marker molecules including *IL-10*, *FOXP3*, *FAP*, *TGFB1*, and *IL-6* and found that the **CRG** risk score is positively correlated with *IL-10* (t = 2.36, *p* = 0.02), *FAP* (t = 3.3, *p* = 1.08e-03), and *TGFB1* (t = 4.25, *p* = 2.75e-05) (Figure 4A; Supplementary Figure S5D). Therefore, we wondered whether the current immunotherapy-related drugs can improve the prognosis of patients in the high-risk group. We investigated the correlation between the **CRG** risk score and the known targets genes of immunotherapy, including *PVR*, *PDCD1*, *CD96*, *TIGIT*, *IDO1*, *CD274*, *CTLA4*, and *LAG3*. Consistent with our predictions, the CRG risk score is positively correlated with *PDCD1* (t = 2.2, *p* = 0.03), *TIGIT* (t = 3.24, *p* = 1.32e-03), *IDO1* (t = 2.11, *p* = 0.04), *CD274* (t = 2.51, *p* = 0.01), *CTLA4* (t = 3.76, *p* = 2.01e-04), and *LAG3* (t = 2.67, *p* = 7.93e-03) (Figure 4B; Supplementary Figure S6D). The DNA methylation of these genes showed no significant changes among these genes in **HCC** (Supplementary Table S3).

## Discussion

Copper is an essential nutrient with redox properties that can be both beneficial and harmful to cells. The role of copper in tumor biology is gradually being recognized and the understanding of cuprotosis in tumors is continuously being improved (Riley et al., 2019). Numerous observations had shown that tumor tissue requires higher levels of copper than healthy tissue (Shanbhag et al., 2021). Gene analysis in clear cell renal cell carcinoma suggested that **CRGs** play a key role in clinical outcomes of this disease (Xia et al., 2017).

For liver cancer, there are currently insufficient studies supporting a role for **CRGs** in this disease. Our study found that the **CRGs** are significantly overexpressed in liver cancer. Among the 13 published **CRGs**, we found that the expression levels of five genes are correlated with the prognosis of liver cancer patients. Except for the high expression of *FDX1*, which indicated a lower risk of poor prognosis, the other genes, *ATP7A*, *DLAT*, *DLD*, and *PDHB*, all correlated with poor prognosis. We constructed a prognostic score model composed of these five genes and found that patients with high **CRG** risk scores tend to have the worse prognosis in all three datasets. FDX1 and fatty acylation of proteins are key factors in copper ionophore-induced cell death (Dorsam and Fahrer, 2016). Deletion of FDX1 blocks the progress of the **TCA** cycle, triggering the accumulation of pyruvate and α-ketoglutarate in cells and promotes tumor development (Rayess et al., 2012). DLAT is one of the components of the pyruvate dehydrogenase (PDH) complex, which catalyzes the decarboxylation of pyruvate in the **TCA** cycle to form acetyl-CoA (Tsvetkov et al., 2022). The expression of ATP7A in breast cancer tissues is significantly higher than that in normal tissues, and inhibiting the expression of ATP7A can improve the sensitivity of breast cancer to cisplatin (Yu et al., 2020). A spectrum of diverse genomic alterations in PDHB has been found in non-clear cell renal carcinoma (Durinck et al., 2015). These research support the significance of our **CRG** model in the prognostic prediction of **HCC**.

The tumor microenvironment is intimately involved the occurrence and development of tumors, and affects the therapeutic effect of any treatments that targets the tumor (Kennedy and Salama, 2020). Several studies have shown that pyroptosis is closely associated with tumor immunity (Gao et al., 2022). In this study, we emphasized the relationship between the immune microenvironment and **CRGs**, and found that in the high-risk group with high expression of **CRGs**, the expression of the type I and II IFN response pathways are significantly lower than that in the low-risk group, indicating that the overall immunity of the patients in the high-risk group is in a suppressed state. In addition, we also found that immunosuppressive Treg cells are significantly increased in the high-risk score group. This suggested that the high expression of **CRGs** can induce immune disorders to promote the development of tumors. The novel immunotherapy agents such as inhibitors of PDCD1, TIGHT, IDO1, CD274, CTLA4 and LAG3 were considered had potential survival benefit in several cancers. The CD274 and PDCD1 immune checkpoint interaction could accelerate cancer progression in the colorectal cancer microenvironment and elderly non-small cell lung cancer patients (Elomaa et al., 2023; Tanaka et al., 2023). The SNP of PDCD1, including rs11568821 and rs2227981 was a prognostic marker in a triple-negative breast cancer (Boguszewska-Byczkiewicz et al., 2023). Moreover, TIGHT regulated TWIST1and promoted vasculature remodeling in bladder cancer (Liu et al., 2022). It also affected autophagy in leukemia and esophageal squamous cell carcinoma (Gschwind et al., 2022; Huang et al., 2023). LAG3 was identified as an important therapeutic target in pancreatic cancer, liver, brain, breast cancer and melanoma (Gulhati et al., 2023; Huuhtanen et al., 2023; Ulase et al., 2023; Zou et al., 2023). In this study, we found that these inhibitors of PDCD1, TIGHT, IDO1, CD274, CTLA4 and LAG3 had potential benefit in high-risk patients.

## Conclusion

With increasing knowledge of the mechanism of copper-driven cell death in tumors, we demonstrated here that this mechanism is also likely to be applicable for **HCC**. Using copper death-related genes, we constructed a prognostic model that will help to better understand the relationship between cuprotosis and liver cancer. The CRG risk score is related to the overall immune status of patients, particularly the presence of Treg cells. This suggested that immune checkpoint inhibitor therapies may have better effects in **HCC** patients with high **CRG** risk scores.

## Figures and Tables

**Table:** 

**TCA**	Tricarboxylic acid cycle
**CRG**	Cuproptosis related genes
**HCC**	Hepatocellular carcinoma
**TCGA**	The cancer genome Atlas
**ICGC**	International cancer genome consortium
**GEO**	Gene expression omnibus
**CNV**	Copy number variation
**SNV**	Single nucleotide variant
**OS**	Overall survival
**LASSO**	Least absolute shrinkage and selection operator
**RS**	Risk score
**ROC**	Receptor operating characteristic
**ssGSEA**	Single-sample gene set enrichment analysis
**AUC**	Area under the curve
**HR**	Hazard ratio
**CI**	Confidence interval
**TNM**	Tumor node metastasis
**AFP**	Alpha fetoprotein
**BCLC**	Barcelona clinic liver cancer
**ALT**	Alanine amiotransferase
**HBV**	Hepatitis B virus
**aDC**	Activated dendritic cell
**APC**	Antigen presenting cell
**CCR**	Cytokine‐cytokine receptor
**HLA**	Human leukocyte antigen
**iDC**	Immature dendritic cell
**pDC**	Plasmacytoid dendritic cell
**Tfh**	T follicular helper cell
**TIL**	Tumor infiltrating lymphocyte
**Treg**	Regulatory T cells
**NK**	Natural killer

## Data Availability

The original contributions presented in the study are included in the article/Supplementary Material, further inquiries can be directed to the corresponding author.
