# Supplementary material for: A novel prognostic prediction model of cuprotosis-related genes signature in hepatocellular carcinoma
Source: Front Cell Dev Biol. 2023 Aug 7;11:1180625. doi: 10.3389/fcell.2023.1180625 (PMC10440422; doi:10.3389/fcell.2023.1180625)
Supplement: Supplementary file 1 [file DataSheet7.PDF]

Table S2

Supplementary Table 2, Primers of CRGs

ATP7A	**Sequence** (5' -> 3')
Forward Primer	TGACCCTAAACTACAGACTCCAA
Reverse Primer	CGCCGTAACAGTCAGAAACAA
DLAT	**Sequence** (5' -> 3')
Forward Primer	CCGCCGCTATTACAGTCTTCC
Reverse Primer	CTCTGCAATTAGGTCACCTTCAT
DLD	**Sequence** (5' -> 3')
Forward Primer	CTCATGGCCTACAGGGACTTT
Reverse Primer	GCATGTTCCACCAAGTGTTTCAT
FDX1	**Sequence** (5' -> 3')
Forward Primer	TTCAACCTGTCACCTCATCTTTG
Reverse Primer	TGCCAGATCGAGCATGTCATT
PDHB	**Sequence** (5' -> 3')
Forward Primer	AGTGGTGGTGCTAGAGAATGA
Reverse Primer	TGCAGCTTCTAAGCAGTGGC
GAPDH	**Sequence** (5' -> 3')
Forward Primer	CTGACTTCAACAGCGACACC
Reverse Primer	TGCTGTAGCCAAATTCGTTGT
