# Supplementary figures and images for: A novel prognostic prediction model of cuprotosis-related genes signature in hepatocellular carcinoma
Source: Front Cell Dev Biol. 2023 Aug 7;11:1180625. doi: 10.3389/fcell.2023.1180625 (PMC10440422; doi:10.3389/fcell.2023.1180625)

A Figure S2

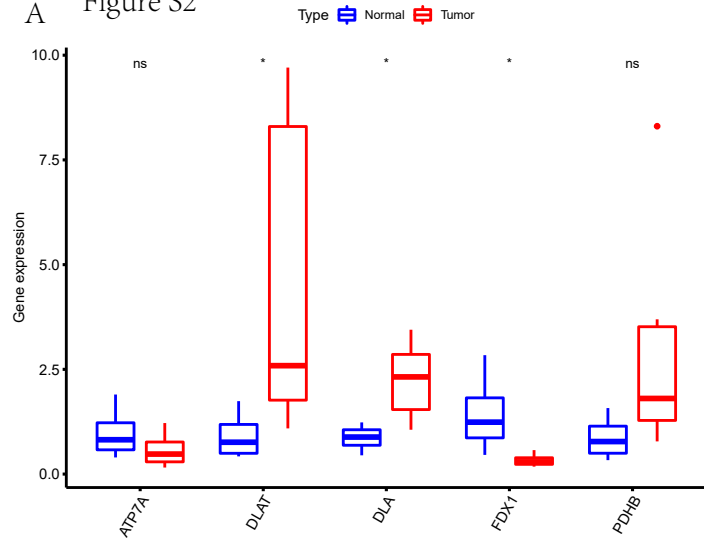

B

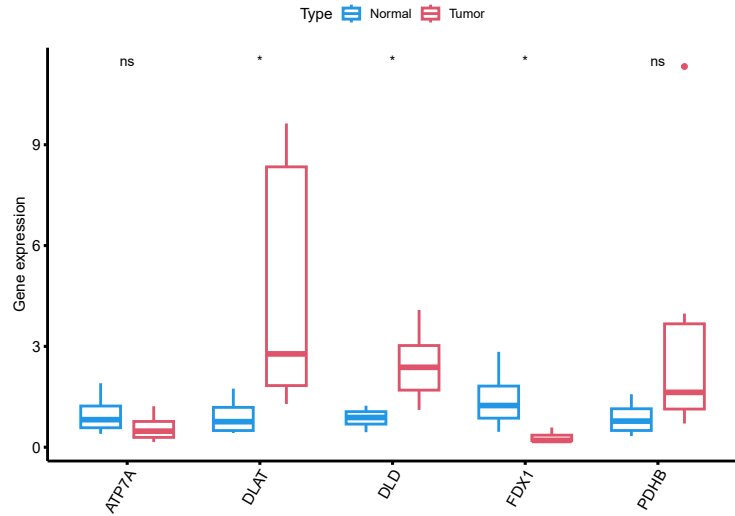

Supplement: Supplementary file 2 [file DataSheet2.PDF]

Figure S4

**A**

**LIRI-JP**

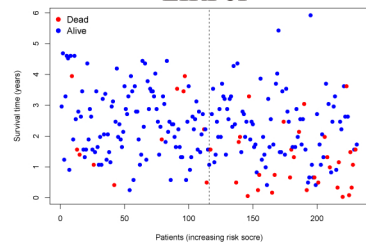

**B**

**GSE14520**

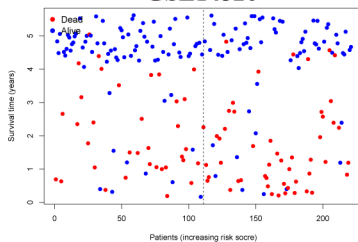

**C**

**TCGA-LIHC**

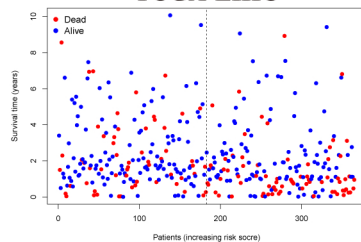

**D**

**LIRI-JP**

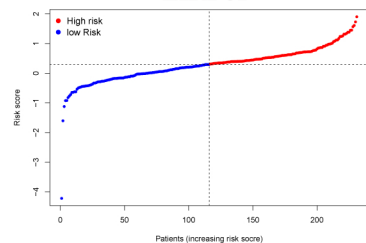

**E**

**GSE14520**

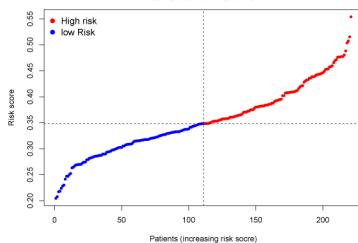

**F**

**TCGA-LIHC**

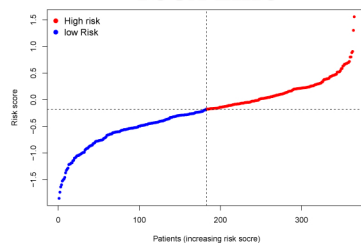

**G**

**LIRI-JP**

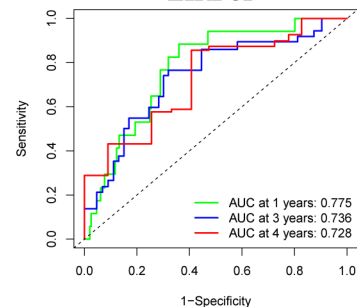

**H**

**GSE14520**

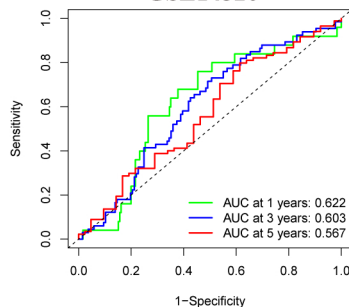

**I**

**TCGA-LIHC**

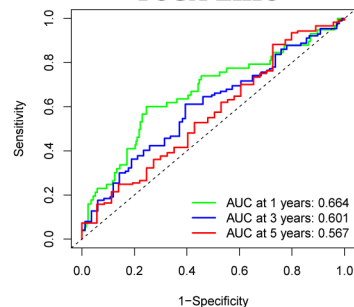

Supplement: Supplementary file 4 [file DataSheet4.PDF]

Figure S6

A

## LIRI-JP

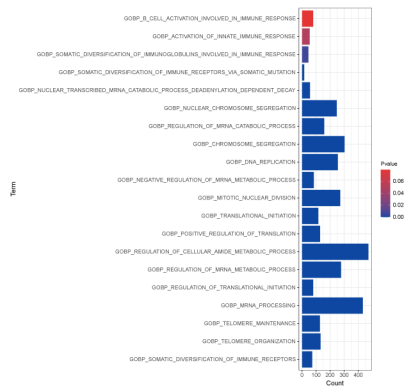

B

## GSE14520

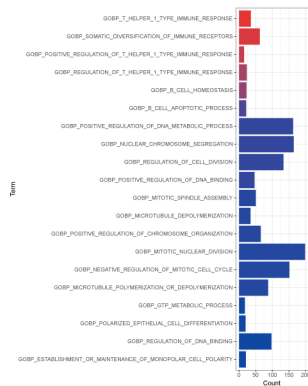

C

## TCGA-LIHC

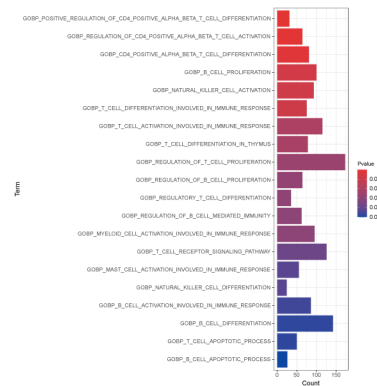

D

## TCGA-LIHC

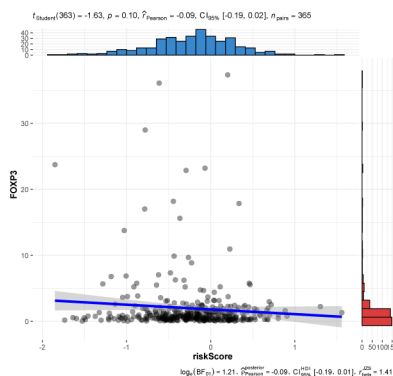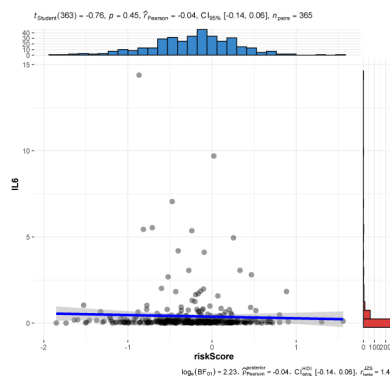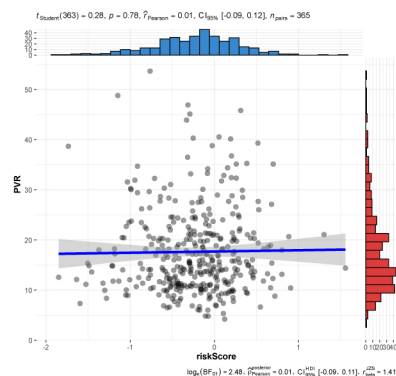

Supplement: Supplementary file 5 [file DataSheet6.PDF]

Figure S3

**A**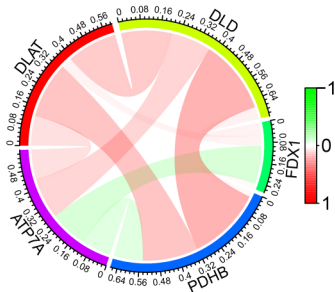**B**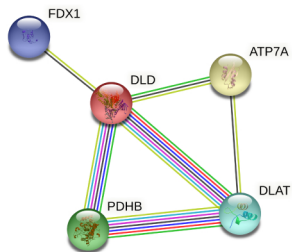**C**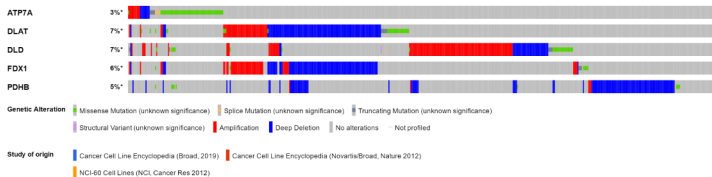**D**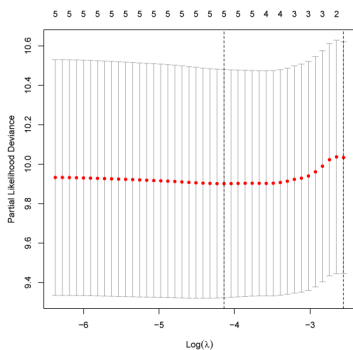**E**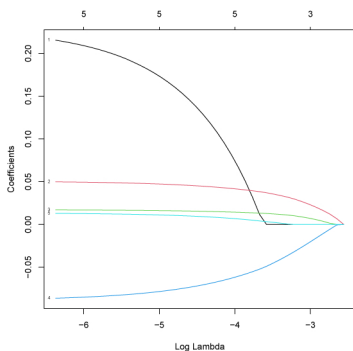

Supplement: Supplementary file 7 [file DataSheet3.PDF]

Figure S1  
A

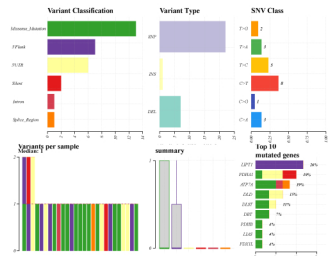

B

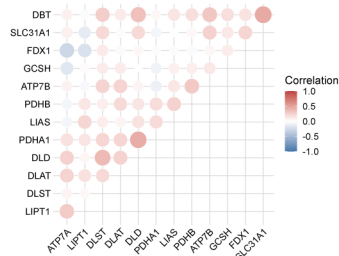

C

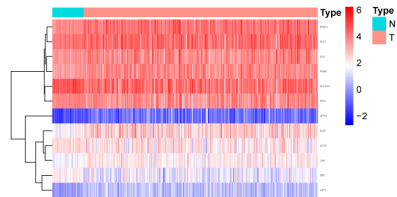

D

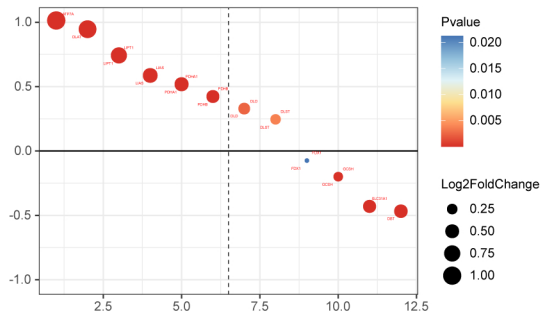

E

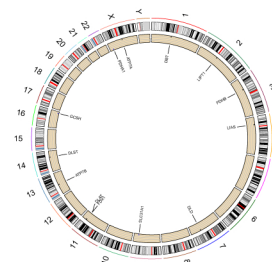

Supplement: Supplementary file 8 [file DataSheet1.PDF]

Figure S5

A

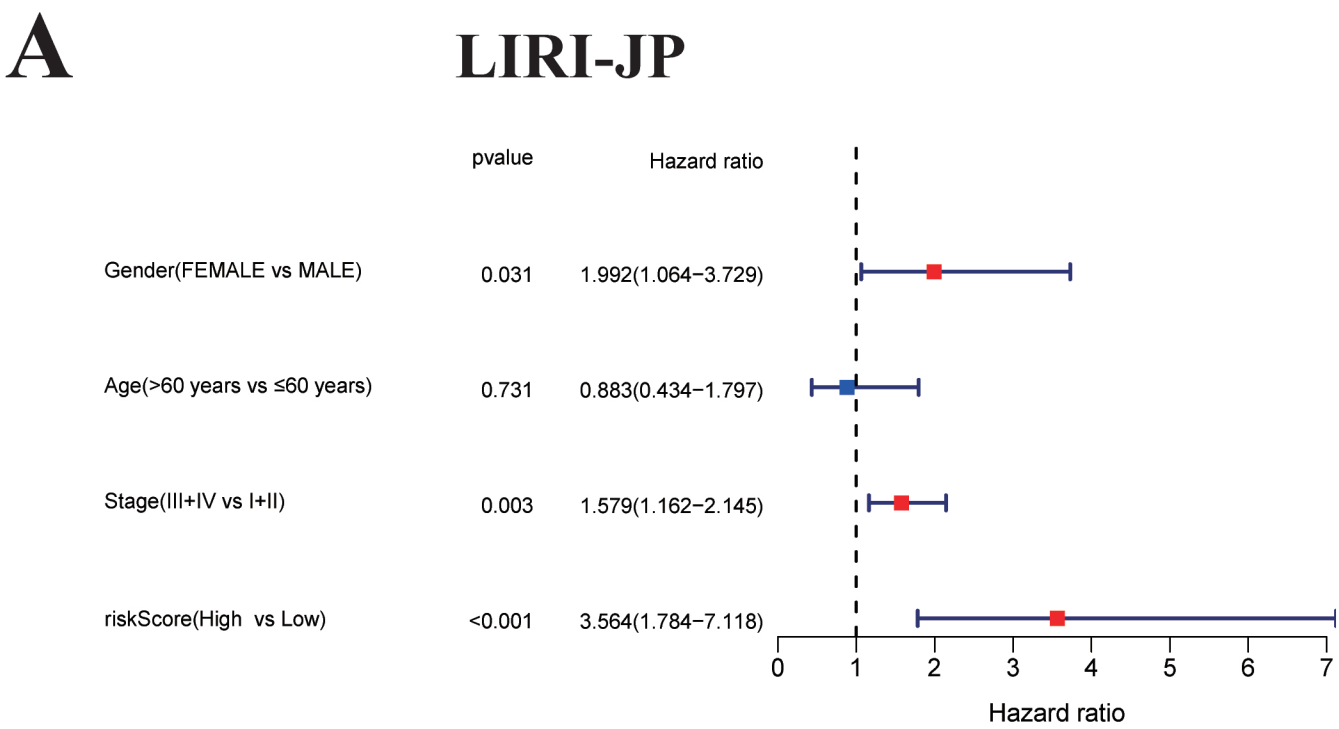

B

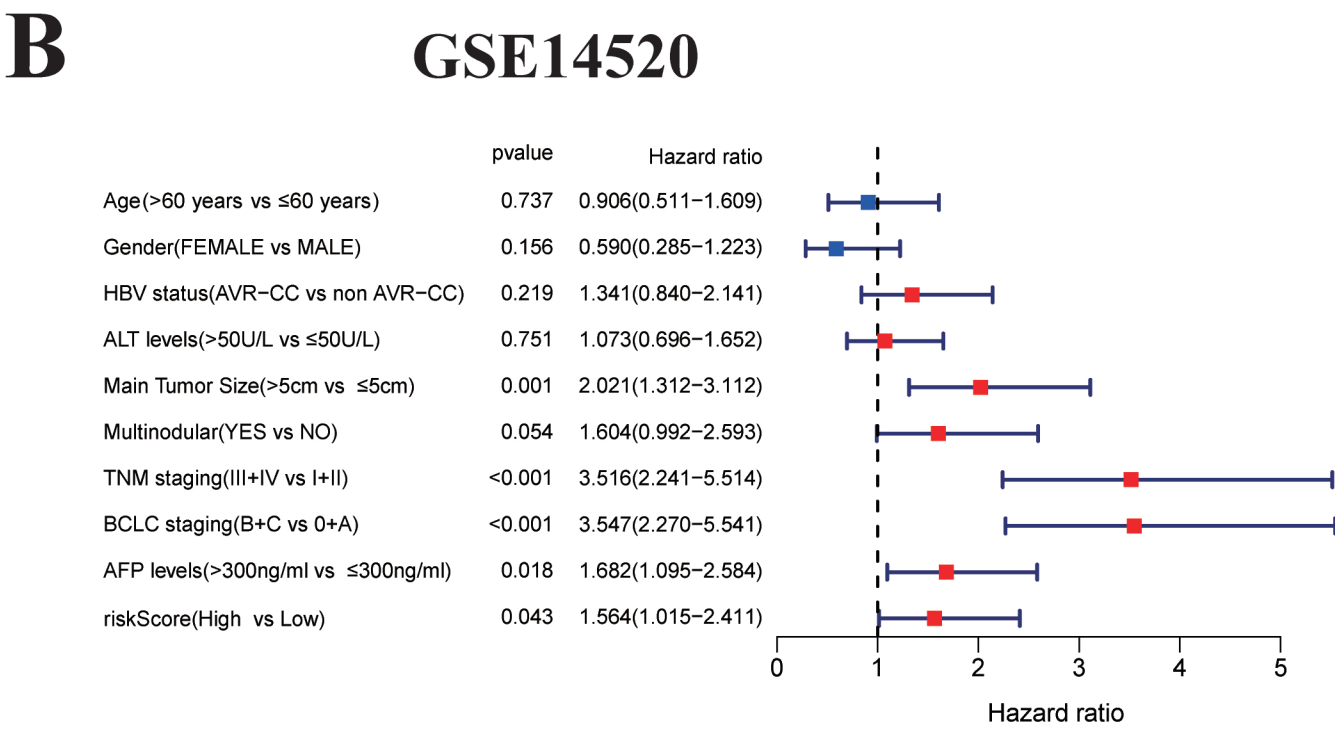

C

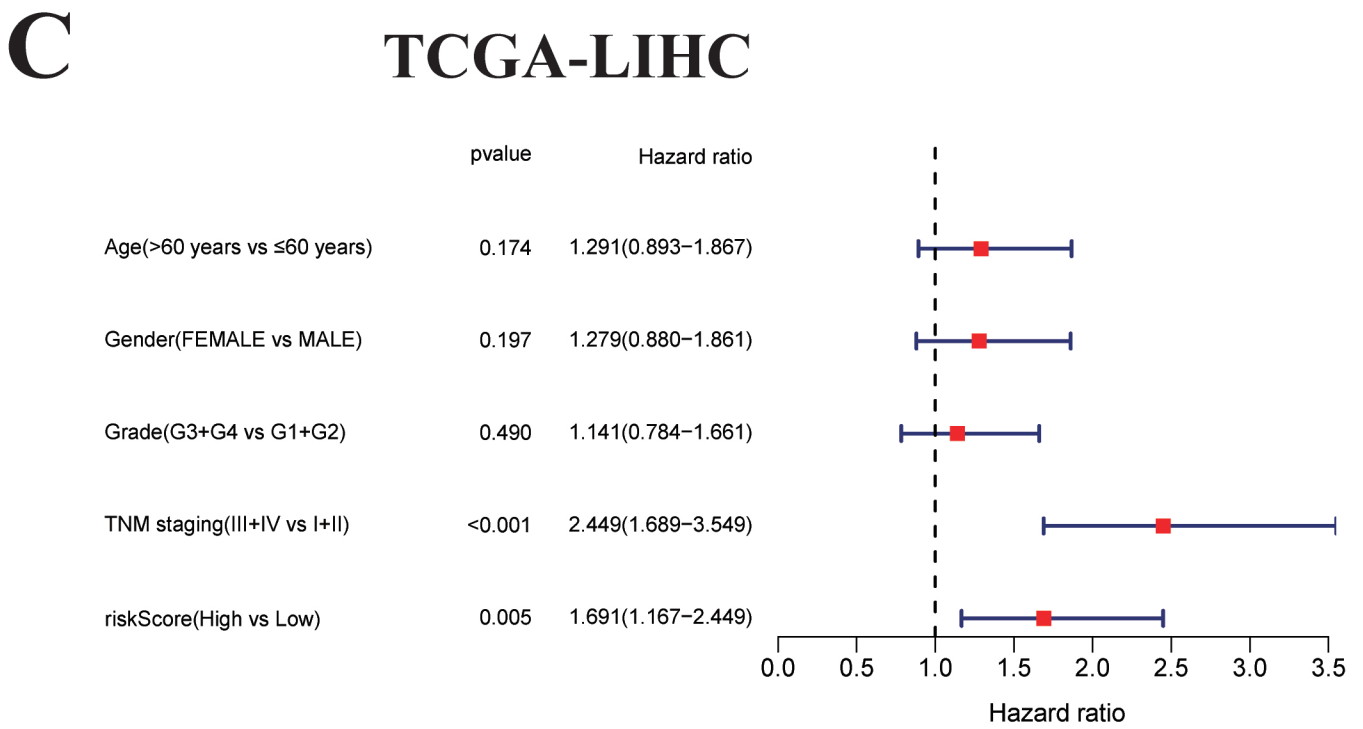

D

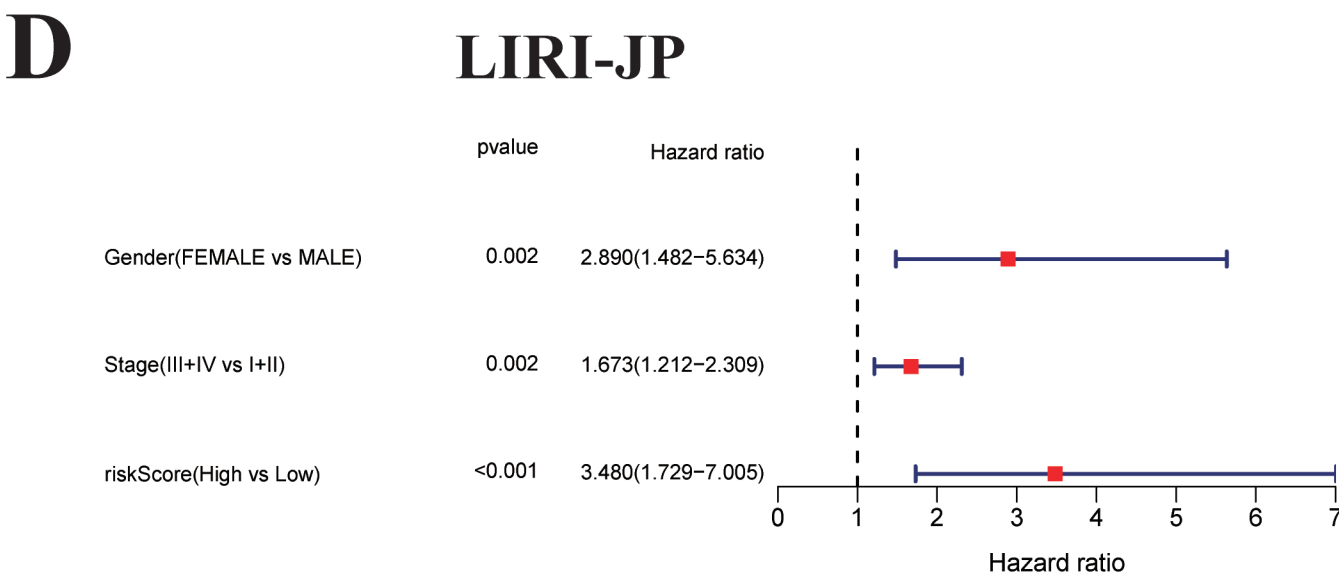

E

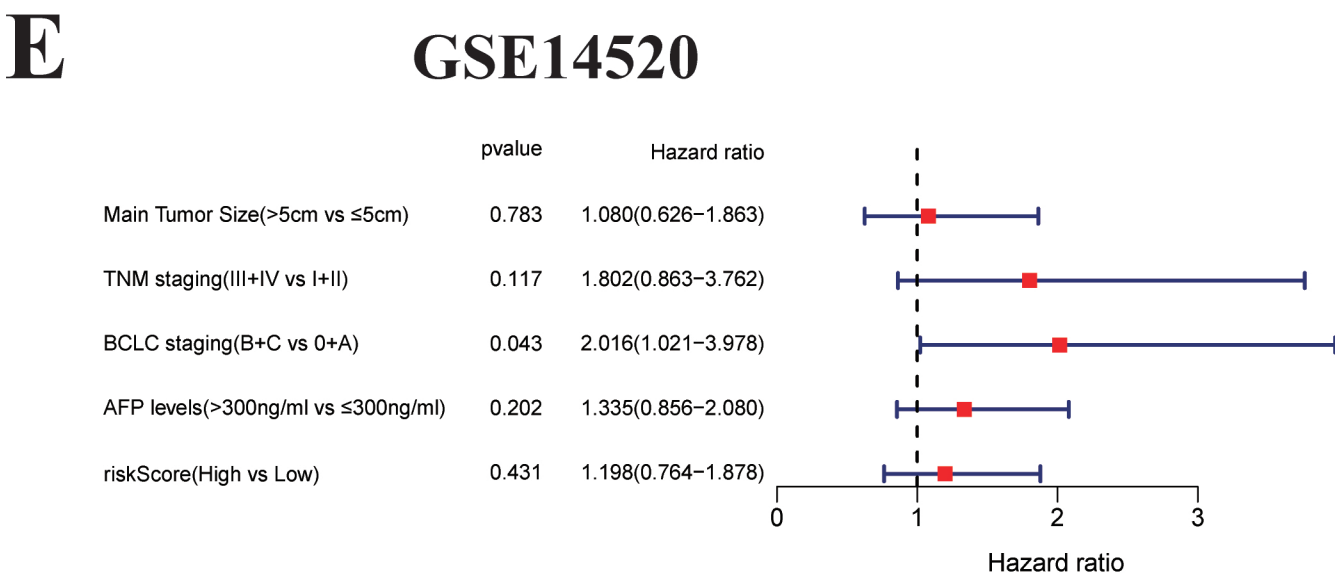

F

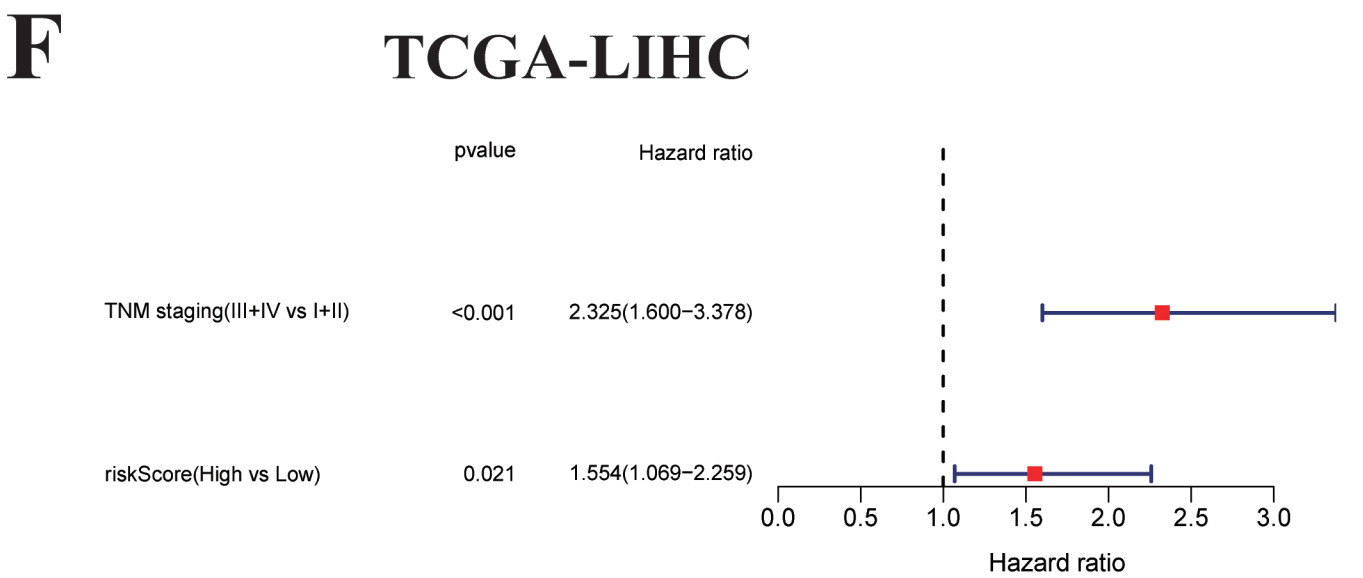

G

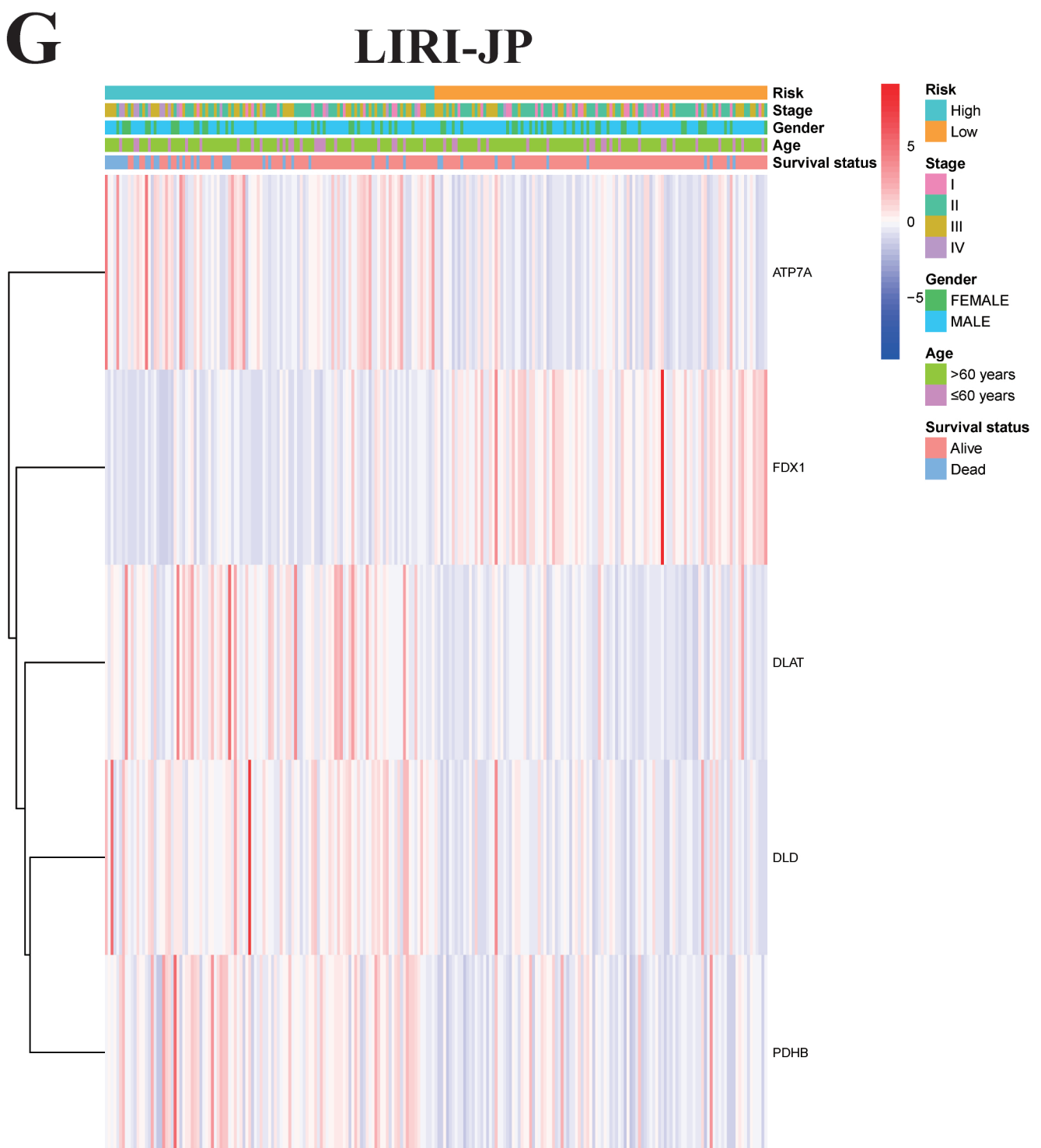

H

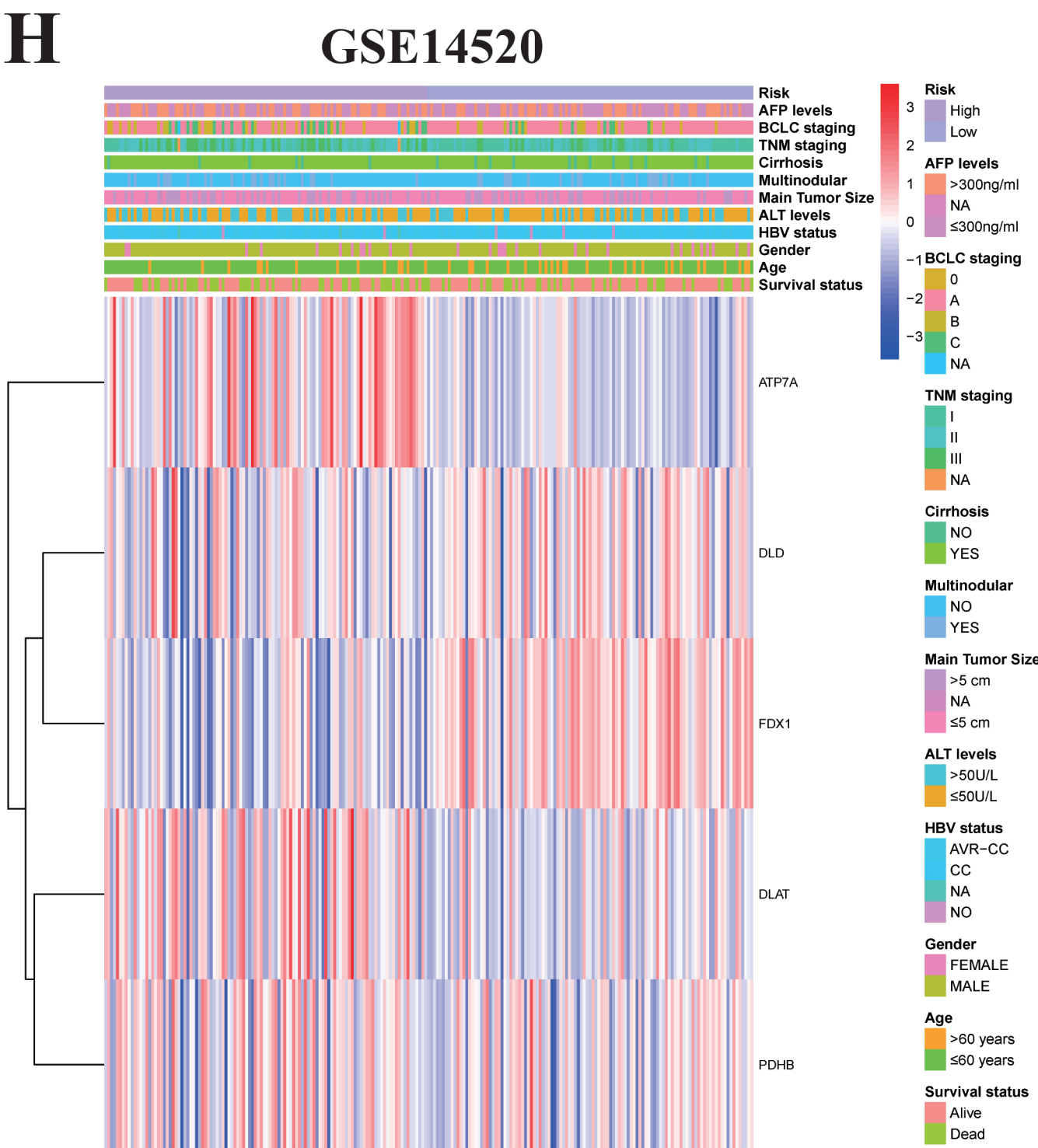

I

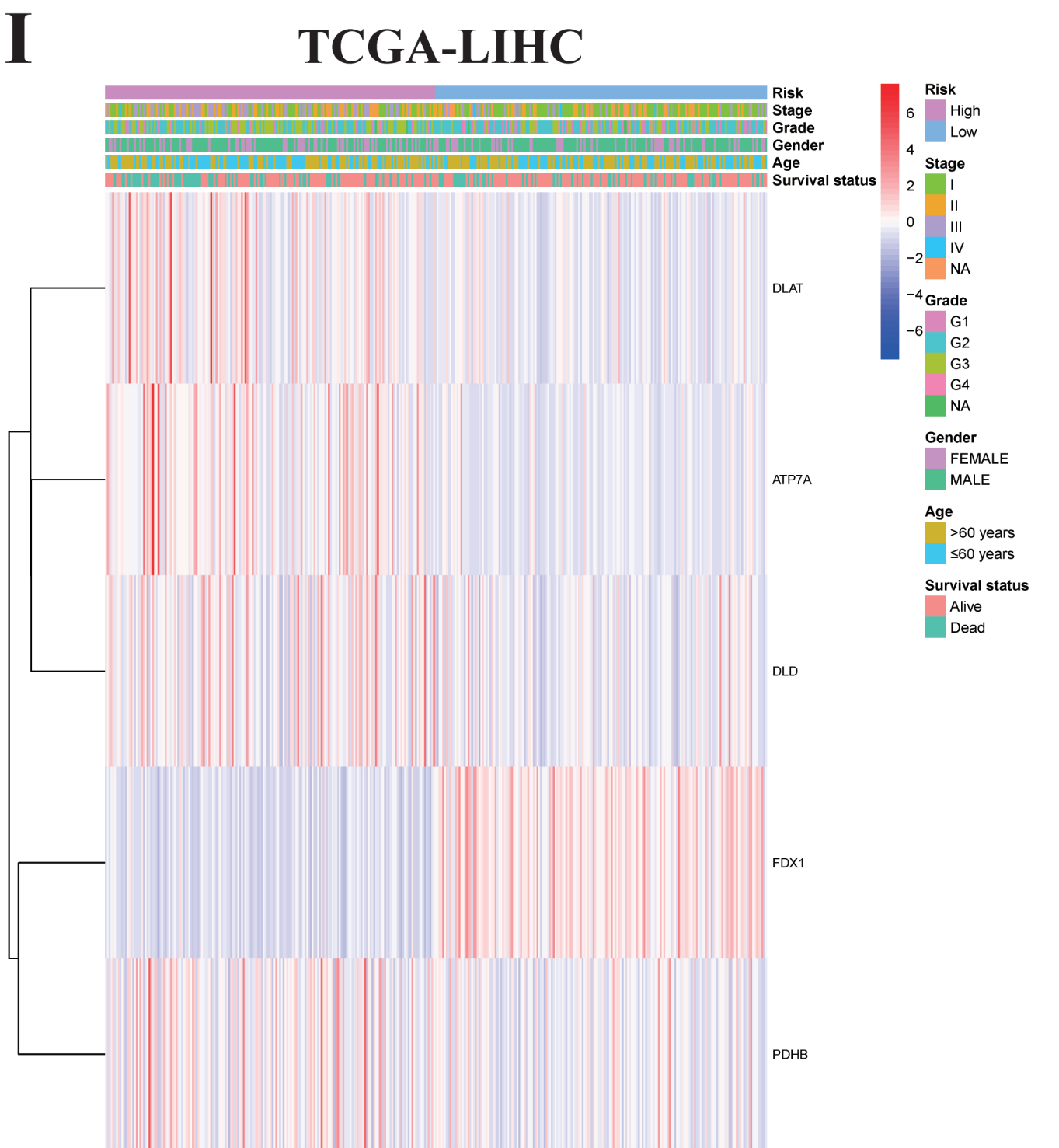

Supplement: Supplementary file 10 [file DataSheet5.PDF]
