# Supplementary material for: A novel prognostic prediction model of cuprotosis-related genes signature in hepatocellular carcinoma
Source: Front Cell Dev Biol. 2023 Aug 7;11:1180625. doi: 10.3389/fcell.2023.1180625 (PMC10440422; doi:10.3389/fcell.2023.1180625)
Supplement: Supplementary file 9 [file Table5.DOCX]

**Supplementary Figure Legend**

Figure 1S, Genetic landscape of CRGs, (A) Mutation status of 13 CRGs in the TCGA database. (B) Expression correlation of CRGs in TCGA database. (C) Heatmap of differential expression of 12 differentially expressed CRGs in TCGA database. (D) Differential expression P value of 12 differentially expressed CRGs in TCGA database. (E) Chromosomal localization of these CRGs.

Figure 2S, Expression of CRGs in cell lines of liver cancers.

Figure 3S, Genetic landscape and prognostic significance of CRGs in HCC. (A) Correlation of five prognosis-related CRGs in ICGC database. (B) The five prognostic CRGs correspond to protein interactions. (C) Mutation status of five prognostic CRGs in CCLE. (D-E) Five prognosis-related CRGs were modeled in the ICGC LIRI-JP dataset as a training set.

Figure S4, Prognostic significance of CRGs in HCC. (A-C) The distributions of OS status, OS and risk score in ICGC, GSE14520 and TCGA cohort. (D-F) The distribution and median value of the risk scores in ICGC, GSE14520 and TCGA cohort. (G-I) AUC of time-dependent ROC curves verified the prognostic performance of the risk score in ICGC, GSE14520 and TCGA cohort.

Figure S5, Implications of CRGs risk score for clinical features and prognosis. (A-C) Results of the univariate Cox regression analyses regarding OS in ICGC, GSE14520 and TCGA cohort. (D-F) Results of the multivariate Cox regression analyses regarding OS in ICGC, GSE14520 and TCGA cohort. (G-I) Heatmap of CRGs risk score for clinical features in ICGC, GSE14520 and TCGA cohort.

Figure S6, Functional analysis and immunoassay of CRGs in HCC. (A-C) GO analysis between high-risk group and low-risk group. (D) Immune-related functions between different risk groups in ICGC, GSE14520 and TCGA cohort.

Supplementary Table 1, Summary of 13 cuproptosis related genes.

Supplementary Table 2, Primers of CRGs.
