# Supplementary material for: A novel prognostic prediction model of cuprotosis-related genes signature in hepatocellular carcinoma
Source: Front Cell Dev Biol. 2023 Aug 7;11:1180625. doi: 10.3389/fcell.2023.1180625 (PMC10440422; doi:10.3389/fcell.2023.1180625)
Supplement: Supplementary file 12 [file Table4.DOCX]

https://www.jianguoyun.com/p/DbXsqFgQzoP_ChiB-_gEIAA
